# Morphological and molecular characterization of a new species of leech (Glossiphoniidae, Hirudinida): Implications for the health of its imperiled amphibian host (*Cryptobranchus alleganiensis*)

**DOI:** 10.3897/zookeys.378.6545

**Published:** 2014-02-07

**Authors:** William A. Hopkins, William E. Moser, David W. Garst, Dennis J. Richardson, Charlotte I. Hammond, Eric A. Lazo-Wasem

**Affiliations:** 1 Dept. of Fish and Wildlife Conservation, Virginia Tech, 106 Cheatham Hall, Blacksburg, VA 24061, USA; 2 Dept. of Invertebrate Zoology, National Museum of Natural History, Smithsonian Institution, Museum Support Center, MRC 534, 4210 Silver Hill Road, Suitland, MD 20746 USA; 3 School of Biological Sciences, Quinnipiac University, 275 Mt. Carmel Avenue, Hamden, CT 06518 USA; 4 Division of Invertebrate Zoology, Peabody Museum of Natural History, Yale University, P.O. Box 208118, New Haven, CT 06520, USA

**Keywords:** Hellbender, leech, disease, parasite, Hirudinida, Glossiphoniidae

## Abstract

The hellbender (*Cryptobranchus alleganiensis*) is among the most intriguing and imperiled amphibians in North America. Since the 1970s and 80s, western populations of the Ozark and eastern subspecies in Missouri have declined by nearly 80%. As a result of population declines, the Ozark hellbender was recently federally protected as an endangered species, and the eastern subspecies was granted protection under CITES. Although habitat degradation is probably the biggest threat to hellbender populations, recent evidence suggests that pathogens including chytrid fungus and “flesh-eating” bacteria may also contribute to declines in Ozark hellbenders. Leeches, which are very common on Ozark hellbenders, have recently been implicated as possible vectors of disease among Ozark hellbenders but have not been described in eastern hellbenders or outside of Missouri and Arkansas. We discovered a population of leeches on eastern hellbenders in southwest Virginia and confirmed that the species of leech is within the genus *Placobdella*, but is morphologically and genetically distinct from all previously described leech species. We named the new species *Placobdella appalachiensis* **sp. n.** Moser and Hopkins, based on the mountainous region in which it was discovered. Our surveys over a three consecutive year period suggested that this leech species may be patchily distributed and/or have a narrow geographic range. We consistently detected leeches at one site (mean prevalence in 80 hellbenders = 27.5%; median intensity = 3.0 leeches per parasitized hellbender [range 1 – >250 leeches]) during three years of surveys, but we never found leeches in any of our other seven study sites in two streams (mean prevalence in 139 hellbenders = 0%). We found a significant positive relationship between hellbender body size and the intensity of parasitism, and we suggest the possibility that the behavioral ecology of adults leading up to reproduction may increase their encounter rates with parasites. We discuss the potential conservation implications of discovery of leeches in this stream, and make recommendations for future mitigation and monitoring efforts.

## Introduction

The hellbender (*Cryptobranchus alleganiensis*) is among the most intriguing and threatened amphibians in North America. This giant salamander reaches total lengths of at least 74 cm (Petranka 1998) and is long-lived (exceeding 30 years, Taber et al. 1975). It is fully aquatic and spends its entire lifecycle within cool, well-oxygenated streams. Occurrence of juvenile and adult hellbenders is strongly associated with large, stable boulders or bedrock used for cover, feeding, and reproduction (Nickerson and Mays 1973; Bodinof 2010). Across the species’ range, which includes the Ozark (*Cryptobranchus alleganiensis bishopi*) and the eastern (*Cryptobranchus alleganiensis alleganiensis*) subspecies, population declines have been documented, and degraded habitat quality appears to play an important role in precipitating declines (Briggler et al. 2007a; Fobes 1995; Mayasich et al. 2003; Trauth et al. 1993). Recent evidence suggests that in addition to habitat degradation, other factors such as disease may also contribute to population declines (Bodinof 2010; Bodinof et al. 2011, 2012; Briggler et al. 2007a, b; Hiler et al. 2005; Trauth et al. 1992). For example, integumental pathogens have recently been documented in wild and captive Ozark hellbenders including the fungus *Batrachochytrium dendrobatidis*, which has been implicated in amphibian declines around the world (Bodinof 2010; Bodinof et al. 2011, 2012; Briggler et al. 2007a, b; Briggler et al. 2008; Lips et al. 2006; Skerrat et al. 2007), and potential “flesh-eating” bacteria that cause grotesque skin lesions (Jeff Briggler, Missouri Dept. Conservation, pers comm.). Widespread declines in the Ozark hellbender have generally been more severe than in the eastern hellbender (Briggler et al. 2007b; Foster et al. 2009; Wheeler et al. 2003), but some populations of eastern hellbenders are known to have declined by more than 80% since the 1970s and 80s (Wheeler et al. 2003). As a result, the Ozark hellbender is now federally endangered and the eastern hellbender has been granted protection under the Convention on International Trade in Endangered Species of Wild Fauna and Flora (CITES) Appendix III (USFWS 2011a, b).

It was recently postulated that leeches may transmit pathogens among amphibians, including Ozark hellbenders (Raffel et al. 2006; Stead and Pope 2010; Huang et al. 2010). Because leeches can influence growth and survival of amphibians (Berven and Boltz 2001) and are ecologically important vectors of organisms such as trypanosomes, malarial parasites, and pathogenic fungi (Sawyer 1986; Mock 1987; Barta and Desser 1989; Raffel et al. 2006), it is important to better understand their relationships with declining amphibians like the hellbender. Leeches were first documented on Ozark hellbenders in the 1960s (Dundee and Dundee 1965; Nickerson and Mays 1973), but these leeches were not formally described as a unique species until Johnson and Klemm (1977). This leech is now recognized as *Placobdella cryptobranchii* (Rhynchobdellida: Glossiphoniidae) (Moser et al. 2006, 2008, 2013). *Placobdella cryptobranchii* has traditionally been thought to be an Ozark hellbender specialist (but see Briggler and Moser 2008; Moser et al. 2013), and has only been collected from the Ozark hellbender subspecies in a small geographic area that includes the North Fork of the White, White, Spring, Current, and Eleven Points Rivers in Arkansas and Missouri, USA (Moser et al. 2006, 2008). Within its isolated geographic range, this small parasite (up to ~ 1.5 cm total length) is very common in populations of Ozark hellbenders, parasitizing the majority of individuals in the population (Nickerson and Mays 1973; Johnson and Klemm 1977; Solís et al. 2007; Moser et al. 2008). Importantly, eastern hellbenders from nearby streams in Missouri are not known to harbor *Placobdella cryptobranchii* or any other species of leech (Huang et al. 2010; J. Briggler, pers. comm.). In fact, only one leech has ever been reported in the literature on an eastern hellbender, but the leech species was not identified (Solís et al. 2007). In light of the fact that hellbenders are declining and that diseases may play a role in declines, understanding the prevalence of potential disease vectors is important for hellbender conservation efforts.

In this study we provide the first thorough documentation of leeches using eastern hellbenders as hosts, in a population far beyond the previously known range of leech infestations in Ozark hellbenders. Using a combination of morphological and molecular techniques, we identify this leech as a new distinct species. Based on three years of field surveys, we documented the prevalence and intensity of leech parasitism in two streams in the Upper TN River basin of Virginia. Given the importance of leeches as vectors of disease-causing organisms, we discuss the potential implications for the health and conservation of hellbenders.

## Materials and methods

### Site description

**Figure 1.**
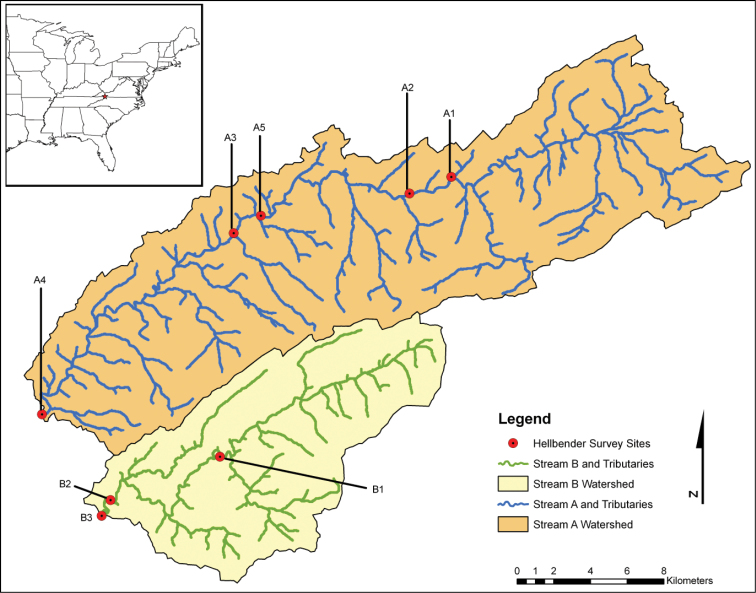
Map of two streams in southwest Virginia, USA where hellbenders were collected from 8 stream reaches over 3 consecutive years. Leeches were only detected in stream reach A3.

The two streams we surveyed in this study are located in the Tennessee River Basin and are part of survey efforts to understand the health and abundance of hellbenders in Virginia, USA (Figure 1). Because of the sensitive status of this species, we refer to the streams as Stream A and B. Both streams drain predominantly (> 70%) forested watersheds and still harbor relatively large populations of hellbenders. However, Stream A is increasingly subjected to a wide range of surrounding land use, including agriculture and development that threatens in-stream water and microhabitat quality.

Surveys for hellbenders were conducted for three consecutive summers in 2007–2009. In the first two years of our surveys, we collected hellbenders from 7 stream reaches (Figure 1), each 100 m long. Habitat characteristics and water quality for these reaches are reported elsewhere (Hopkins and DuRant 2011; Hopkins et al. 2011). After discovering leeches in one reach (A3) in Stream A (see results), we returned to that stream in 2009 and surveyed the reach (A3) containing parasitized hellbenders and an upstream reach (A1) where leeches had not been detected, but we expanded our search efforts in these two reaches to 500 m. In addition, we added an additional eighth stream reach (A5) located 2.1 km upstream from reach A3 to determine if leeches could be detected in close upstream proximity to the site.

### Hellbender surveys

We collected hellbenders during diurnal surveys by turning rocks while skin-diving, which is the best method for obtaining all age classes of hellbenders (Humphries and Pauley 2005; Nickerson et al. 2003; Nickerson and Krysko 2003). We collected all hellbenders between June and the first week of September each year. We conducted our study during mid/late summer because it represents the beginning of the breeding season in these streams, when male and female adult hellbenders can be distinguished by the swollen cloaca of males (Makowsky et al. 2010). However, we completed our study before oviposition typically starts in these streams (September) to avoid disturbing active nests.

Once we captured each hellbender, we transported it to the stream bank for processing. We measured total and snout-vent length (TL and SVL), weighed, and sexed (based on cloacal morphology of adults) each individual and subjected them to a physical exam that included enumeration and removal of leech specimens (see below). We then injected a passive integrative transponder tag (PIT tag) into the tail of each hellbender for future identification and released each individual under the rock where it was initially collected.

We counted and noted the location of leeches on hellbenders before removing them from each hellbender. Leeches were gently secured flat between a cover slip and a glass slide before being relaxed using dropwise additions of 10% ethanol. Once relaxed, a subset of leeches was preserved in 95% ethanol and another subset was fixed in 10% buffered formalin before being preserved in 70% ethanol. The one exception to this protocol was a hellbender collected in 2007 that hosted so many leeches (> 250) that we were unable to count all of them due to constraints of our surveys (see results). In 2008 an additional subset of leeches were held alive in cold stream water to allow them to digest their blood meal before being shipped live or prepared as described above. Four specimens were pressed, stained with Semichon’s acetocarmine, and mounted in Canada balsam for examination by light microscopy according to techniques outlined by Richardson (2006), as modified by Richardson and Barger (2006). Terminology for plane shapes follows Clopton (2004). Specimens were deposited in the Invertebrate Zoology collections of the National Museum of Natural History, Smithsonian Institution (USNM) Washington, District of Columbia and the Peabody Museum of Natural History (YPM), Yale University, New Haven, Connecticut.

### DNA analyses

We conducted molecular analyses on newly collected material according to Richardson et al. (2010). We isolated DNA from the caudal suckers of individual leeches with the DNeasy Blood & Tissue Kit from Qiagen (Cat. No. 69504), following the protocol for purification of total DNA from animal tissues (spin-column). For the proteinase K treatment step, we lysed tissue samples overnight at 56 °C. DNA was eluted from the spin columns with 150 µl of buffer.

We prepared PCR Reactions using the Illustra PuRe Taq Ready-To-Go PCR beads from GE Health Care (Cat. No. 27-9559-01). Primers were purchased from Invitrogen and were comprised of 2 primers each for cytochrome c oxidase subunit I (CO-I) as specified by Light and Siddall (1999). Specifically the CO-I primers were LCO1490 (5’GGTCAACAAATCATAAAGATATTGG 3’) and HCO2198 (5’TAAACTTCAGGGTGACCAAAAAATCA 3’). The final volume of PCR reactions was 25 µl with 2 µl of leech genomic DNA added per reaction. We amplified DNA under the following PCR conditions: 94 °C for 5 min.; 35 cycles of (94 °C for 30 sec, 50 °C for 30 sec, 72 °C for 45 sec); 72 °C for 7 min. Following PCR, samples were purified using a QIAquick PCR purification kit from Qiagen (Cat. No. 28104). Purified PCR products were sequenced using the HCO2198 primer and the LCO1490 primer for the cytochrome c oxidase subunit I products by the W. M. Keck Foundation Biotechnology Resource Laboratory at Yale University. We aligned DNA sequences using Clustal W version 2 (Larkin et al. 2007) and checked them manually using SeaView 4 (Gouy et al. 2010). We then analyzed them using PAUP* 4.0b10 (Swofford 2002) and compared them to other leech DNA sequences contained within Genbank.

### Statistical analyses

We ran all statistical analyses in SAS 9.1 (SAS Institute Inc., Cary, NC, USA) or Microsoft Excel and recognized statistical significance at α < 0.05. Where appropriate, we tested for normality and homoscedasticity using Ryan-Joiners and Bartlett’s tests, respectively. Unless otherwise noted, we used raw data in statistical analyses.

Because leeches were only detected in one stream reach (see results), we did not statistically compare the incidence of leech parasitism across streams or reaches. Within the site where leeches were common, we compared prevalence (% of individuals harboring at least one leech) using a Fisher’s exact test. The intensity of leech infestation (number of leeches per parasitized individual) was compared among the three years using a Kruskal Wallis test because data were not normally distributed and transformation did not improve the distribution. Among individuals that were parasitized, we used linear regression to determine whether there was a relationship between body size (total length) and the number of leeches attached to individuals. We used a Chi-Square test and a Kruskal Wallis test to evaluate whether sex of adults differed in their probability of being parasitized or the number of leeches they harbored, respectively. We excluded the one individual with > 250 leeches from comparisons of size and sex because we did not have a precise count of leeches on this individual and it was a clear outlier compared to the rest of the population.

## Results

### Survey results

In total, we captured 219 hellbenders at our eight sites over the three year study. All age classes were detected in the two streams, from gilled larvae to large adults. Body mass of hellbenders ranged from 2.0 to 1,040 g, total length ranged from 6.2–58.2 cm, and snout-vent length ranged from 4.1 to 37.1 cm.

**Figure 2.**
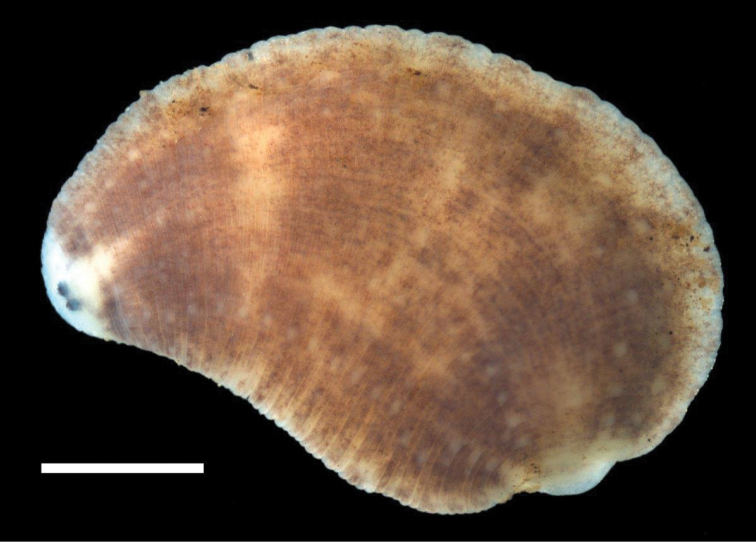
Dorsal surface of *Placobdella appalachiensis* sp. n., Holotype USNM 1232924 collected from an adult eastern hellbender (*Cryptobranchus alleganiensis*) from stream reach A3 in southwest Virginia, USA. Scale bar equals 1 mm.

**Figure 3.**
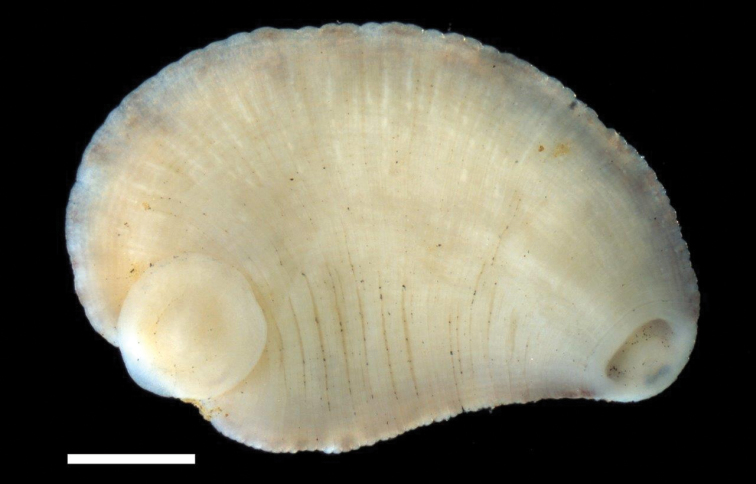
Ventral surface of *Placobdella appalachiensis* sp. n., Holotype USNM 1232924 collected from an adult eastern hellbender (*Cryptobranchus alleganiensis*) from stream reach A3 in southwest Virginia, USA. Scale bar equals 1 mm.

**Figure 4.**
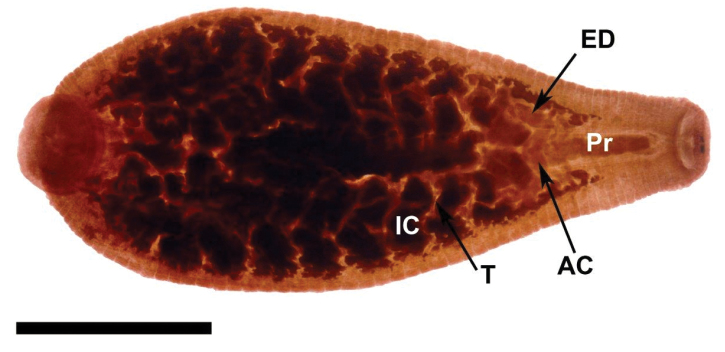
Internal anatomy of *Placobdella appalachiensis* sp. n., Paratype USNM 1232939 collected from an adult eastern hellbender (*Cryptobranchus alleganiensis*) from stream reach A3 in southwest Virginia, USA. Ventral view, atrial cornuae (**AC**), ejaculatory duct (**ED**), intestinal ceca (**IC**), proboscis (**Pr**), testisac (**T**). Scale bar equals 2 mm.

We only detected leeches in stream reach A3, where they tended to be quite common (Table 1). No leeches were detected at any other sites, including nearby reach A5 which was added in 2009 to determine the upstream extent to which leeches could be detected. The species of leech was determined to be within the genus *Placobdella*, but was morphologically and genetically distinct from all species described to date (see Species Description below (Figures 2–4).

**Table 1.** Prevalence and intensity of parasitism of eastern hellbenders by the leech *Placobdella appalachiensis* sp. n. in Stream Reach A3 in southwest Virginia, USA. No leeches were found in the other 7 stream reaches over 3 years of study. Prevalence represents the percentage of hellbenders parasitized by at least one leech. Intensity of parasitism is calculated as the number of leeches on parasitized individuals.

Parameter	2007	2008	2009	All YRS COMBINED
	Prevalence			
Sample size	17	16	47	80
# Parasitized	6	6	10	22
Prevalence (%)	35.3	37.5	21.3	27.5
	Intensity			
Sample Size	6	6	10	22
Mean Intensity	44.8	13	6.6	18.8
Median Intensity	2.5	3.5	3.0	3.0
Range	1 – >250	1 – 40	1 – 24	1 – >250

**Figure 5.**
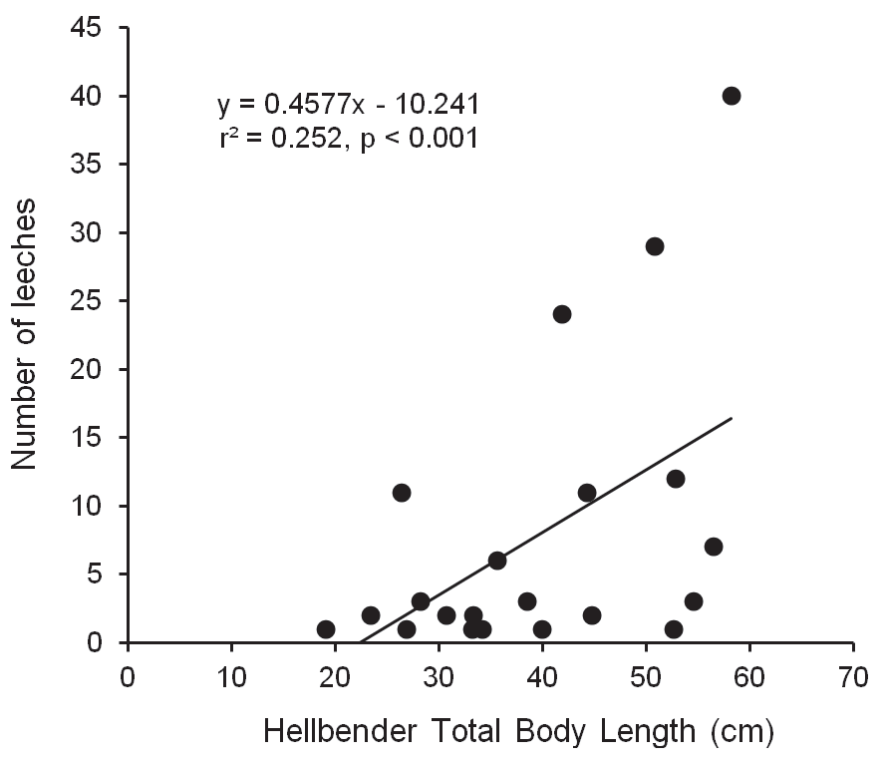
Relationship between total body length (cm) of eastern hellbenders (*Cryptobranchus alleganiensis*) and the number of leeches (*Placobdella appalachiensis* sp. n.) they harbored. All hellbenders were collected from stream reach A3 in southwest Virginia, USA.

Within the site (A3) where leeches were prevalent, we found leeches on diverse size classes of hellbenders ranging from juveniles (19.1 cm TL) to large adults (58.2 cm TL). Leeches were primarily found on dorsal surfaces of the head, torso, and tail, but also on limbs and the gular region of the throat. Prevalence of leech parasitism was fairly consistent among years, ranging from 21.3–37.5% (p = 0.332). Likewise, median intensity of parasitism ranged from 2.5–3.5 leeches per parasitized hellbender among the three years (p = 0.942). There was a significant positive relationship between body size of hellbenders and the number of leeches they harbored, when all individuals (r^2^ = 0.12, p < 0.001) and when only parasitized individuals (r^2^ = 0.25, p < 0.001; Figure 5) were included in the model. Adult males and females (N = 22 and 26, respectively) were equally likely to harbor leeches (mean prevalence = 36.4% and 34.6%, respectively; p = 0.90). Of the individuals with leeches, adult males tended to harbor more leeches than females but there was no significant difference between the sexes (mean intensity = 38.1 and 10.0, respectively; median intensity = 6.5 and 2.0, respectively; p = 0.331).

Several hellbenders harbored very small (< 1–2 mm) leeches in all three years, providing evidence that leeches are successfully reproducing at the site and that hatchlings were likely using hellbenders for their first blood meal. In fact, one adult that we collected in 2007 harbored > 250 leeches, of which at least 200 were small hatchlings and juveniles. Of the 80 hellbenders collected at reach A3, 13 were recaptures in the second and/or third year of the study. There were no obvious patterns of parasite attachment across years for individuals that were recaptured. Five recaptured individuals did not harbor leeches in any of their capture years. Four individuals were not parasitized during their first year, but were parasitized when recaptured. In contrast, four individuals harbored fewer leeches in later years than in earlier captures, but these four individuals should be interpreted carefully since we removed voucher specimens from all parasitized hellbenders.

### Species description
Family Glossiphoniidae Vaillant, 1890

#### Placobdella appalachiensis

Moser & Hopkins sp. n.

http://zoobank.org/33FF4843-BB57-4682-9D8F-8B2745BF57F0

http://species-id.net/wiki/Placobdella_appalachiensis

Figs 2 – 4

##### Material examined.

Holotype (USNM 1232924) South Fork Holston River, Smyth County, Virginia.

Paratypes (USNM 1232925 – 1232942; YPM IZ 067799) South Fork Holston River, Smyth County, Virginia.

##### Morphological description.

*External morphology*. Body very deeply to deeply ovoid and triannulate. Length of preserved specimens 2.5 – 10.4 mm long, mean + SE 5.2 + 0.4 mm (n = 23), width at widest point (in posterior half of body) 2.0 – 5.9 mm, mean + SE 3.4 + 0.2 mm (n = 23). Dorsum chocolate (#7B3F00) to russet (#80461B) brown with 6 rows of papillae (2 para-medial, 2 para-lateral, & 2 lateral) and many, thin, unpigmented, vertical lines (Figure 2; see hex color codes http://www.colorhexa.com). Para-medial, para-lateral, and lateral papillar rows begin at two points (just lateral to the anus) and the papillae are on the neural annulus. Apical cephalic region unpigmented, extending and tapering posteriorly through one thin nuchal band. Two pairs of eye spots (one pair much larger than the other) within cephalic unpigmented region, and occasionally separated by a little less than the diameter of the larger eyespot. Anal region and small genital bar unpigmented to diffusely pigmented with scattered unpigmented to diffusely pigmented small patches in between. Caudal sucker, 0.5 – 2.1 mm in diameter, mean + SE, 1.2 + 0.1 (n = 18), generally unpigmented with a few brown chromatophores and without papillae. Ventrum unpigmented with very sparse scattering of a few brown/green chromatophores (Fig. 3). Male and female gonopores in furrows and separated by 2 annuli.

##### Internal morphology.

*Digestive system*: Proboscis posteriad of the rim/lip of the oral sucker. Short, blunt-tipped proboscis, uniformly cylindrical, and in membranous sheath. Salivary cells scattered in the anterior third of the body (diffuse salivary glands) and slightly more abundant near the base of the proboscis. Salivary ductule bundles join with retractor muscles and attach at each side of the base of the proboscis. Flaccid esophagus extends from the base of the proboscis with one pair of saccular mycetomes. Seven pair of diverticulated crop ceca and the last pair extending posteriad into four sections and diverticulated. Four pair of simple, saccular intestinal ceca. Simple rectum opening to anus, located one annulus anteriad of the caudal sucker.

*Reproductive system*: (Male) Male gonophores slightly raised. Male atrium opening into ovoid or elloptoid atrial cornue extending laterally and anteriorly from male gonopore into robust, coiled, muscular ejaculatory ducts, recurving posteriorly to robust seminal vesicles and narrow vas deferentia connecting to testisacs. Six pairs of orbicular testisacs, each testisac located in the space between pair of crop ceca. (Female) Female gonopore simple, opening to pair of bifurcated ovisacs. Ovisac length depends on the reproductive condition of the leech.

##### Taxonomic summary.

Type host: Eastern Hellbender, *Cryptobranchus alleganiensis alleganiensis* (Daudin, 1803).

##### Type locality.

South Fork Holston River, Smyth County, Virginia.

##### Type material.

USNM 1232924 (Holotype), USNM 1232925 – 1232942 (Paratypes), YPM IZ 067799 (Paratype).

##### Etymology.

Named for the Appalachian Region, where the leech is known to occur.

##### Molecular description.

DNA Analysis.

Molecular comparison of 637 nucleotides of CO-I revealed differences of 0.2% to 1.3% (1–8 nucleotides) among four specimens of *Placobdella appalachiensis* sp. n. (GenBank KF990590–KF990593) collected from South Fork Holston River, Smyth County, Virginia. Differences of 17.7% to 19.1% (113 to 122 nucleotides) were found between four specimens of *Placobdella appalachiensis* sp. n. and seven specimens of *Placobdella cryptobranchii* (GenBank KF601755–KF601761) collected from Missouri. CO-I sequence data among four specimens of *Placobdella appalachiensis* sp. n. revealed differences of 18.7% to 19.6% (119 to 125 nucleotides) compared to five specimens of *Placobdella ornata* (GenBank JQ8128–JQ8132) collected from the type locality (West River, New Haven County, Connecticut), differences of 18.8% to 20.0% (120–127 nucleotides) compared to four specimens of *Placobdella ornata* collected from the type locality (Shivericks Pond, Falmouth, Barnstable County, Massachusetts) of *Placobdella phalera* (junior synonym of *Placobdella ornata*) (GenBank JQ812133–JQ812136), differences of 17.7% to 18.7% (113–119 nucleotides) compared to two specimens of *Placobdella translucens* (GenBank AY047328, JX122778), differences of 15.8% to 16.8% (101–107 nucleotides) from 1 specimen of *Placobdella picta* (GenBank AF116020), differences of 17.2% to 18.1% (110–115 nucleotides) from 1 specimen of *Placobdella biannulata* (GenBank AF116021), and differences of 17.9% to 19.0% (114–121 nucleotides) from 2 specimens of *Placobdella sophieae* (GenBank KF990594–KF990595) collected from Oregon.

## Discussion

Our study provides the first comprehensive description of a population of eastern hellbenders (*Cryptobranchus alleganiensis alleganiensis*) parasitized by leeches. We identified the leech as a new species, *Placobdella appalachiensis*, based on its distinct morphological and genetic characteristics. Importantly, leeches were only detected in one reach of the rivers we studied, suggesting that the population of leeches is either isolated or that they are patchily distributed within the river(s). Given the fact that leeches can transmit pathogens amongst individuals, our discovery may have important implications for hellbender conservation (Davis and Hopkins 2013).

Ozark hellbenders (*Cryptobranchus alleganiensis bishopi*) are commonly parasitized by a congener, *Placobdella cryptobranchii*, in several rivers in Missouri and Arkansas (Johnson and Klemm 1977; Moser et al. 2008, 2013). The closest location of a population of *Placobdella cryptobranchii* (Eleven Point River) is approximately 850 kilometers from the type locality of *Placobdella appalachiensis*. Although these leeches utilize the similar host species (*Cryptobranchus alleganiensis bishopi* and *Cryptobranchus alleganiensis alleganiensis*, respectively) molecular comparison of CO-I sequence data from *Placobdella cryptobranchii* revealed differences of 17.7% to 19.1% with *Placobdella appalachiensis* sp. n. In addition to these molecular differences, *Placobdella appalachiensis* sp. n. is also distinguished from *Placobdella cryptobranchii* based on external morphology. *Placobdella appalachiensis* has a chocolate (#7B3F00) to russet (#80461B) brown dorsum with many thin unpigmented vertical lines, 6 rows of papillae, one unpigmented nuchal band, unpigmented to sparsely pigmented patches, and no pre-anal papillae. In contrast, *Placobdella cryptobranchii* recently redescribed by Moser et al. (2013), has a rust, reddish-brown dorsum with 2 lateral rows of unpigmented papillae, two unpigmented nuchal bands, unpigmented patches, and four pair of pre-anal papillae.

*Placobdella appalachiensis* sp. n. shares morphological similarities with several other leeches. For example, it is also morphologically similar to *Placobdella sophieae* (Oceguera-Figueroa et al. 2010) and *Placobdella picta* (Verrill, 1872). *Placobdella sophieae* also has 6 rows of dorsal papillae, but it is near transparent green and does not possess a nuchal band or unpigmented to sparsely pigmented patches (Oceguera-Figueroa et al. 2010) as found in *Placobdella appalachiensis*. Additionally, *Placobdella sophieae* is only known from its type locality of northern Washington (Oceguera-Figueroa et al. 2010). *Placobdella picta* has been sporadically reported in the southeastern United States, including Virginia (Klemm 1982; 1985), typically in woodland ponds or lakes (Sawyer 1972; Barta and Sawyer 1990). *Placobdella picta* has a nuchal band and 6 to 7 rows of papillae, but also has a thin dark dorsal medial line and has a brown-green dorsal coloration variegated with orange (Barta and Sawyer 1990), which are not found in *Placobdella appalachiensis*. Another amphibian-leech occurring in the southeastern United States is *Placobdella biannulata* (Moore, 1900). *Placobdella biannulata* does not have an unpigmented nuchal band or genital and anal patches and is further distinguished from *Placobdella appalachiensis*, by the former’s olive green dorsum and biannulate body segments (Moser et al. 2005a). Comparison of *Placobdella appalachiensis* COI sequence data to these three congenerics revealed differences of 15.8% to 19.0%.

Leeches were common on hellbenders within the stream reach where they were detected. Overall, 27.5% of individuals collected in reach A3 harbored leeches, with a median intensity of infestation of 3 leeches per individual. However, the prevalence of parasitism we observed was substantially lower than that observed by *Placobdella cryptobranchii* on Ozark hellbenders in Missouri and Arkansas. In a survey of the North Fork White, Spring, and Eleven Points Rivers, 71% of sampled hellbenders hosted 1-140 leeches, with a mean infestation of 8.7 leeches per individual (Moser et al. 2008). Likewise, Nickerson and Mays (1973) and Johnson and Klemm (1977) noted that as many as 96% of Ozark hellbenders they sampled in Missouri and Arkansas were parasitized by *Placobdella cryptobranchii* and Solís et al. (2007) found that ≥ 50% of the Ozark hellbenders they sampled in Missouri harbored leeches.

The fact that we only found leeches at one study reach within these two streams raises questions about the detectability and geographic distribution of this new species. The widespread prevalence of the Ozark hellbender leech makes it readily detectable within its previously known geographic range in Missouri and Arkansas (Dundee and Dundee 1965; Johnson and Klemm 1977; Nickerson and Mays 1973; Solís et al. 2007; Moser et al. 2008). In contrast, our results suggest that the leech species we discovered occurs in lower abundance and/or is patchily distributed, which might make it more difficult to detect in populations of eastern hellbenders. Hellbenders have been well surveyed in other eastern states including North Carolina, Georgia, West Virginia, Tennessee, and Pennsylvania (e.g., Foster et al. 2009; Humphries and Pauley 2005; Petokas et al. 2006; Horchler 2010). It seems probable that given these survey efforts, *Placobdella appalachiensis* would have been detected if it was widespread and abundant. However, it is also possible that its small body size and relatively cryptic morphology contribute to its lack of detection. In the only published account to date of a leech on an eastern hellbender, Solís et al. (2007) mentioned a single leech attached on an eastern hellbender from either North Carolina or Georgia (not specified in the paper), but this leech was not identified and voucher specimens were not deposited. However, several recent accounts (e.g., Dale McGinnity per comm., 2012) provided evidence that leeches occur in Tennessee, but it is currently unknown whether this leech was *Placobdella appalachiensis*. Further work is needed to describe the distribution, local abundance, and natural history of this new species of leech in Appalachia.

Compared to previous studies examining leeches in Ozark hellbenders, ours is the first to detect a significant influence of hellbender body size on the intensity of parasitism by leeches. However, Raffel et al. (2006) found that larger red spotted newts (*Notophthalmus viridescens*) harbored a larger number of a closely related leech species (*Placobdella picta*) than smaller newts and attributed their findings to accrual of parasites with age. In our study, it seems likely that other factors besides continual accrual might influence our observed relationship between hellbender size and parasitism, especially because members of this leech genus are known to detach seasonally. For example, it is possible that microhabitat use, movement patterns, or social interactions of larger adults during the reproductive season puts them at greater risk of encountering leeches than subadults and juveniles. At the time of year when we conducted our study, adult hellbenders become more active and males are thought to begin commandeering and excavating nest sites under large flat rocks (Nickerson and Mays 1973). Thus, the reproductive ecology, habitat use, and perhaps even physiology of adults, particularly large adult males, may place them at greater risk of encountering leeches in the environment than smaller individuals. These patterns warrant further study with larger sample sizes than those reported here.

It remains unclear as to whether *Placobdella appalachiensis* sp. n. is an eastern hellbender specialist or whether it uses multiple vertebrate host species. For comparison, the Ozark hellbender leech (*Placobdella cryptobranchii*) is not known to parasitize eastern hellbenders in Missouri and Arkansas where they occur in close geographic proximity to Ozark hellbenders, which is interesting given the similar habitat use and behavior of these two hellbender subspecies. However, Briggler and Moser (2008) suggested that the Ozark hellbender leech may be capable of using multiple hosts after they found a single mudpuppy (*Necturus maculosus*) in the Eleven Point River (Missouri, USA) that harbored four blood-fed *Placobdella cryptobranchii*. They postulated that with the precipitous population decline of its primary host (i.e., Ozark hellbenders), *Placobdella cryptobranchii* may be forced to diversify hosts. However, more recent experimental work suggests that *Placobdella cryptobranchii* are reluctant to attach to mudpuppies in captivity, suggesting that this field observation may have been a rare or isolated event (Moser et al. 2013). Use of multiple host species has been shown in other leeches, and can be influenced by a variety of factors. For example, Moser et al. (2005b) explored the host attachment dynamics of a closely related leech, *Placobdella biannulata*, which specializes on *Desmognathus* salamanders. Based on field surveys and lab studies of several species of amphibians, the authors concluded that host specificity in this system is probably most influenced by ecological and behavioral factors that influence encounter rates with leeches. Additional surveys at our study sites are needed to determine whether the new leech species that we discovered in Virginia uses any other vertebrates as hosts because host specificity may influence the distribution of this leech as well as its potential to serve as a vector of pathogens within and among host species.

Our discovery of a new species of leech using eastern hellbenders as hosts has important implications for the health and conservation of these imperiled salamanders. Leeches influence the growth and survival of amphibians, are important vectors of disease, and have even been implicated as contributors to amphibian population declines. Raffel et al. (2006) provided evidence from field surveys that leeches from the same genus (*Placobdella picta*) serve as the primary means of transmitting *Icthyophonus* sp., a fungus that has caused mass mortality and morbidity events in other amphibian species. In the same study, leeches were also associated with transmission of trypanosomes. Correlative evidence of pathogen transmission is also available for hellbenders. For example, in a recent study on Ozark and eastern hellbenders in seven rivers in Missouri, blood parasites were prevalent in populations where leeches were common, but no blood parasites were detected in populations where leeches were absent (Huang et al. 2010). Further, cytological evidence consistent with parasitic infection was detected in the populations of hellbenders that harbored leeches and blood parasites. Based on these lines of evidence, the authors postulated that leeches may serve as a key vector for blood parasite transmission that may affect the health of hellbenders (Huang et al. 2010). Similarly, recent work by our group has also identified trypanosomes within the same stream reach where we found leeches (Davis and Hopkins 2013). Future efforts in southwest Virginia should build upon this foundational evidence to determine whether leeches may transmit disease-causing organisms among eastern hellbenders and whether this influences the health of these imperiled amphibians. In the process of studying leeches in our system and others, precautions should be taken to prevent foreign leech introductions among streams, as they are commonly introduced around the world when hosts, water, and/or substrate are translocated by humans (Govedich et al. 2002; Siddall and Budinoff 2005; Moser et al. 2005c).

## Supplementary Material

XML Treatment for Placobdella appalachiensis
